# Formononetin and mizoribine inhibit Porcine Reproductive and Respiratory Syndrome Virus replication *in vitro*

**DOI:** 10.3389/fnut.2024.1501685

**Published:** 2025-03-24

**Authors:** Zhanding Cui, Jinlong Liu, Jinlong Wang, Jing Zhang, Yimei Cao, Kun Li, Zhixun Zhao, Hong Yuan, Xingwen Bai, Xueqing Ma, Pinghua Li, Yuanfang Fu, Huifang Bao, Dong Li, Qiang Zhang, Zaixin Liu, Kai Li, Tong Xu, Zengjun Lu

**Affiliations:** ^1^Key Laboratory of Preventive Veterinary Medicine, Department of Veterinary Medicine, Animal Science College, Hebei North University, Zhangjiakou, Hebei, China; ^2^State Key Laboratory for Animal Disease Control and Prevention, College of Veterinary Medicine, Lanzhou University, Lanzhou Veterinary Research Institute, Chinese Academy of Agricultural Sciences, Lanzhou, China; ^3^Institute of Traditional Chinese Veterinary Medicine, College of Veterinary Medicine, Gansu Agricultural University, Lanzhou, China

**Keywords:** Porcine Reproductive and Respiratory Syndrome, formononetin, antiviral agent, mizoribine, swine viral infections

## Abstract

This study delves into the antiviral efficacy of Formononetin (FMN) and Mizoribine (MZR) against the Porcine Reproductive and Respiratory Syndrome Virus (PRRSV), a virus with a considerable economic impact and a current void in effective treatments. FMN and MZR were found to inhibit various PRRSV strains *in vitro*, predominantly in the early stages of viral infection. Noteworthy was the observation of their synergistic effects when combined with Ribavirin. The study underscores the antiviral potential of FMN and MZR, particularly emphasizing their low cytotoxicity at specific concentrations. These results position FMN and MZR as promising antiviral agents against PRRSV, underscoring their low cytotoxicity and efficacy in early-stage viral inhibition. Such findings pave the way for their potential inclusion in future PRRSV management strategies.

## Introduction

The Porcine Reproductive and Respiratory Syndrome Virus (PRRSV) represents a pathogen of considerable economic consequence (1, 2). Globally, PRRSV has escalated to pandemic proportions, pervading regions including North America, South America, Europe, and Asia (3). Annually, it inflicts financial losses surpassing $650 million in the United States (4, 5). Classified within the order *Nidovirales* and family *Arteriviridae*, PRRSV is an enveloped virus characterized by a single-stranded, positive-sense RNA genome (6). The virus typically induces two distinct clinical phases: reproductive failure and respiratory illness, against which current vaccines offer limited efficacy, and no other effective treatments are available (7). Infection with highly pathogenic strains of PRRSV can lead to severe symptoms such as respiratory distress, anorexia, and fever (2, 8). Autopsy findings often include pronounced pulmonary consolidation and interstitial pneumonia (9). Research has shown that PRRSV predominantly targets macrophages in the pulmonary and alveolar regions, resulting in significant pulmonary damage (9).

The PRRSV is characterized by a large genome and a deficiency in effective correction mechanisms, leading to pronounced genomic heterogeneity (10). This heterogeneity fosters significant genetic variation among PRRSV strains, thereby impeding the development of potent vaccines for pandemic control (11). Economically, the virus imposes losses through production constraints and elevated disease management costs (5). More critically, PRRSV infection heightened their vulnerability to other infectious diseases and further intensifies its economic repercussions (3, 12). The virulence of PRRSV strains displays considerable variability across different regions and periods (13). While most PRRSV outbreaks are marked by low morbidity and mortality rates, substantial epidemics still emerge almost every year (14). In such instances, multiple strains may coexist within a single region or farm (15). The absence of efficacious antiviral agents against this consequential virus is a significant concern. Antiviral drugs, serving as a consistent approach to hinder viral replication, could complement vaccines in managing the pandemic (16). In our prior research, we unexpectedly found that formononetin and mizoribine effectively inhibit the replication of *Feline Calicivirus* (FCV) *in vitro* (17, 18). Formononetin (FMN, 7-hydroxy-3-(4-methoxyphenyl)-4H-chromen-4-one) exhibits notable antioxidant, anticancer, and anti-inflammatory properties (19). The chemical structure of mizoribine (MZR, 1-[(2R,3R,4S,5R)-3,4-dihydroxy-5-(hydroxymethyl)oxolan-2-yl]-5-hydroxy-1H-imidazole-4-carboxamide) is characterized by a purine nucleoside analog structure. This configuration allows mizoribine to act as an immunosuppressive agent by inhibiting inosine monophosphate dehydrogenase (IMPDH) and disrupting nucleotide biosynthesis. MZR shares structural similarities with Ribavirin (RBV), a long-standing and broad-spectrum antiviral medication (20).

In this research, we initially confirmed the efficacy of FMN and MZR in inhibiting PRRSV *in vitro*. Further investigations revealed that FMN and MZR are also effective against various other PRRSV strains maintained in our laboratory. Notably, when used in conjunction with RBV, FMN or MZR displayed a synergistic effect in suppressing PRRSV. Time-of-addition studies have shown that both FMN and MZR predominantly target the early stages of PRRSV replication, significantly hindering its proliferation.

## Materials and methods

### Cells, compounds and viruses

#### Compounds and preparation

Formononetin (F141481, Aladdin), Mizoribine (M129842, Aladdin), and Ribavirin (R101754, Aladdin) were procured from Aladdin. Each compound was dissolved in DMSO (D8418, Sigma) to create a 50 mM stock solution.

#### Cell lines

MARC-145 cells, utilized in this study, were obtained from the China Center for Type Culture Collection (Wuhan, China). These cells were cultured in a controlled environment at 37°C with 5% CO_2_, using Dulbecco’s Modified Eagle’s Medium (DMEM; Catalog No. 12491015, Gibco), enriched with 8% fetal bovine serum (FBS; Catalog No. 16140071, Gibco). Porcine Alveolar Macrophages (PAM) cells were isolated and cultured as previously detailed (21).

#### Viruses

The strains GSWW-15 (GenBank accession: KX767091.1) and GSWW-18 (GenBank accession: OP764591.1) were isolated and maintained in our laboratory (22). The Porcine Reproductive and Respiratory Syndrome Virus (PRRSV) VR-2332 strain (GenBank accession: EF536003.1) was sourced from Boehringer Ingelheim.

### Indirect immunofluorescence assay (IFA)

The following antibodies were used for IFA: Mouse Anti-SR30 (Rtilab, SR30-A) and Goat Anti-Mouse FITC (Bioss, bs-0368G-FITC). After virus inoculation, cells were fixed with 80% cold acetone according to previous experimental conditions for IFA (18). A Leica DMI6000 microscope was used for observation.

### Virus titer and RNA expression level

Viral solutions were diluted in a 10-fold gradient. Aliquots of solutions containing varying concentrations of the virus (100 μL each) and DMEM with 2% FBS (100 μL) were added to each well. Add 100 μL of 2% FBS DMEM (without PRRSV) to the control wells. The plates were incubated for 4 days at 37°C in a humidified atmosphere with 5% CO_2_, after which the virus TCID_50_ values were calculated using the Reed and Muench formula. To evaluate PRRSV gene expression, we employed the relative quantitative Reverse Transcription Quantitative Polymerase Chain Reaction (qRT-PCR) technique. The resultant data were processed using Design & Analysis Software (Thermo, version 2.6.0) and normalized based on the 2^−ΔΔCt^ method. For the determination of viral copy numbers, absolute quantitative qRT-PCR was utilized, with sample normalization achieved by quantifying plasmid concentrations prior to the detection process. The specific methods are as described in section 2.4 of our previous paper (18). The upstream and downstream primers were: PRRSV 5’-CTAAGAGAGGTGGCCTGTCG’ and 5’-GAGACTCGGCATACAGCACA-3′; Marc-145-GAPDH 5’-CCTTCCGTGTCCCTACTGCCAAC-3′ and 5’-GACGCCTGCTTCACCACCTTCT-3′; PAM-GAPDH 5’-TCTGGCAAAGTGGACATT-3′ and 5’-GGTGGAATCATACTGGAACA-3′.

### Cytotoxicity and half-maximal inhibitory concentration

Cells were treated in a 96-well plate using DMEM supplemented with 8% FBS. After achieving a monolayer formation, the cells were treated with multiple concentrations of the test compound, which were diluted in DMEM containing 2% FBS and added to six replicate wells. As a control, a blank solution consisting of 0.5% DMSO in DMEM was employed. The cells treated with the compound were incubated at 37°C in a humidified atmosphere with 5% CO_2_ for 48 or 72 h. Subsequently, the half-maximal cytotoxic concentrations (CC_50_) was measured following the protocol described in section 2.2 of our previous work (18). For the determination of the half-maximal effective concentration (EC_50_), each concentration was tested in triplicate wells, using 0.4% DMSO as a blank control. EC_50_ was determined by assessing PRRSV genomic expression levels after three freeze–thaw cycles, and results were plotted using GraphPad Prism 8.

### Interaction assessment of compounds

As mentioned before, the checkerboard method was employed to perform serial dilutions and mixtures of two compounds to determine the combined effect of CAPE and RBV (23). The TCID_50_ values were determined, and the effects of the combination were evaluated using SynergyFinder (24). The ZIP model was used to calculate the synergy scores for combinations of drugs at different concentrations (25).

### Time-of-addition assay

**Figure 1:**
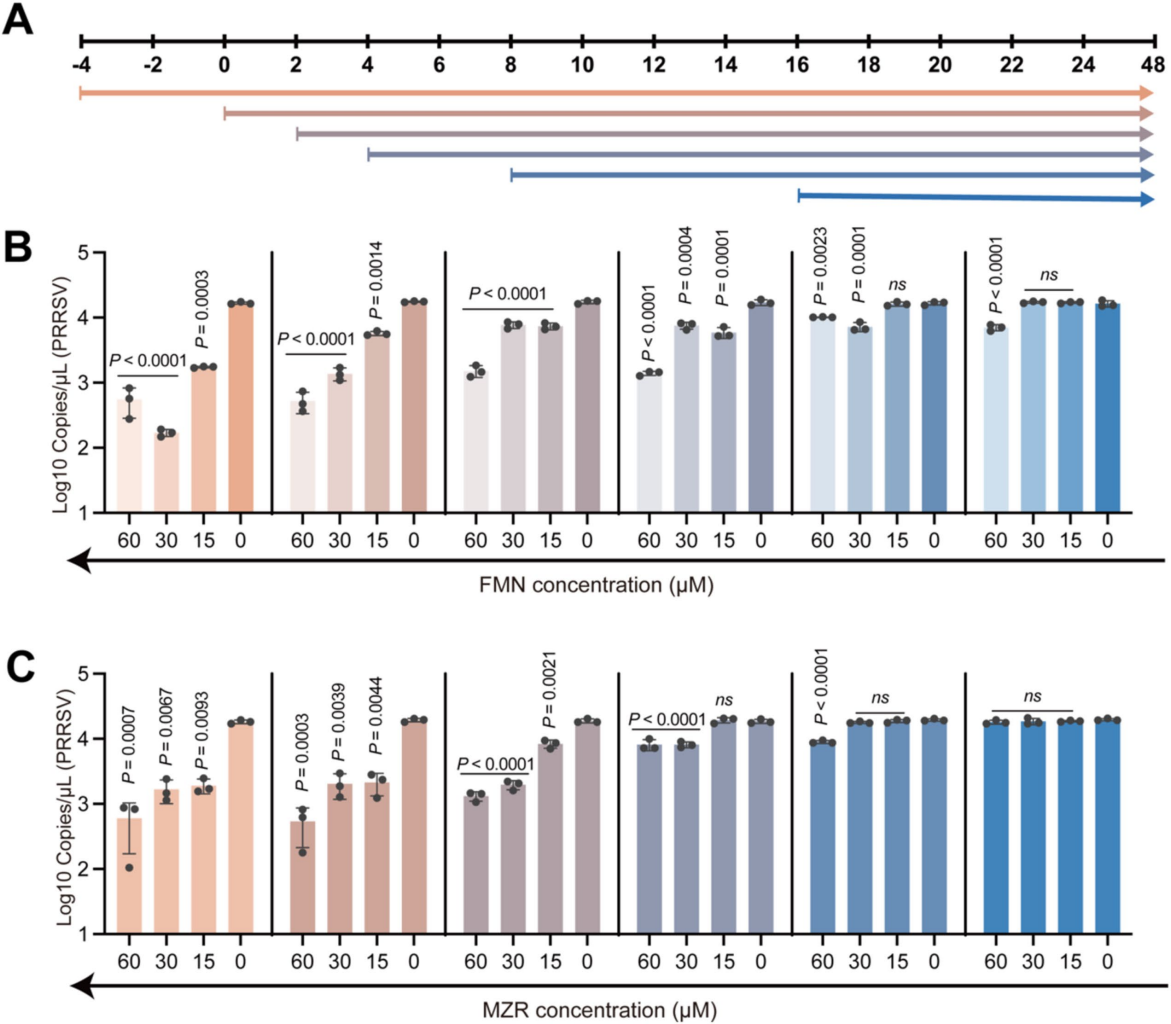
Time-of-addition experiment with FMN or MZR. **(A)** This schematic diagram illustrates the classical experimental protocol for administering drugs at different time points. The experiment began with Marc-145 cells uniformly infected with VR-2332 (MOI = 1) at 0 h, followed by adding varying concentrations of FMN or MZR at specific times. **(B,C)** FMN or MZR was introduced at −4, 0, 2, 4, 8, and 16 h to examine their impact on various stages of PRRSV replication. All samples were collected for analysis 36 h post-infection. The colors of the bars in the figure correspond to the time points as detailed in **(A)**. Each symbol represents an independent biological replicate. The *p*-values are indicated, with ‘ns’ denoting *p* > 0.1234. These values were calculated using one-way ANOVA with Dunnett’s multiple-comparison test, and a *p*-value less than 0.0332 was considered statistically significant.

The assay commenced with seeding cells into a 96-well plate, followed by their cultivation in DMEM supplemented with 8% FBS. Upon achieving a monolayer, the cells were infected with the VR-2332 strain of PRRSV at a multiplicity of infection (MOI) of 1. One hour after infection, the viral inoculum was discarded, and the cells were cleansed with ice-cold PBS. DMSO or various concentrations of FMN or MZR were then introduced to the culture at different time intervals, as depicted in Figure 1A. Thirty-six hours post-infection, the expression levels of PRRSV RNA were quantified using previously described methods.

### Graphs and statistical analysis

Unless otherwise specified, graphical representations and statistical analyses were performed using GraphPad Prism (version 9.5.1). When comparing two variables, a two-tailed or one-tailed unpaired *t*-test was conducted to determine statistical significance. Detailed statistical information for each experiment is described in the figure legends. The final figures were assembled in Adobe Illustrator (CS6 version).

## Results

### Cytotoxicity test of FMN and MZR

**Figure 2:**
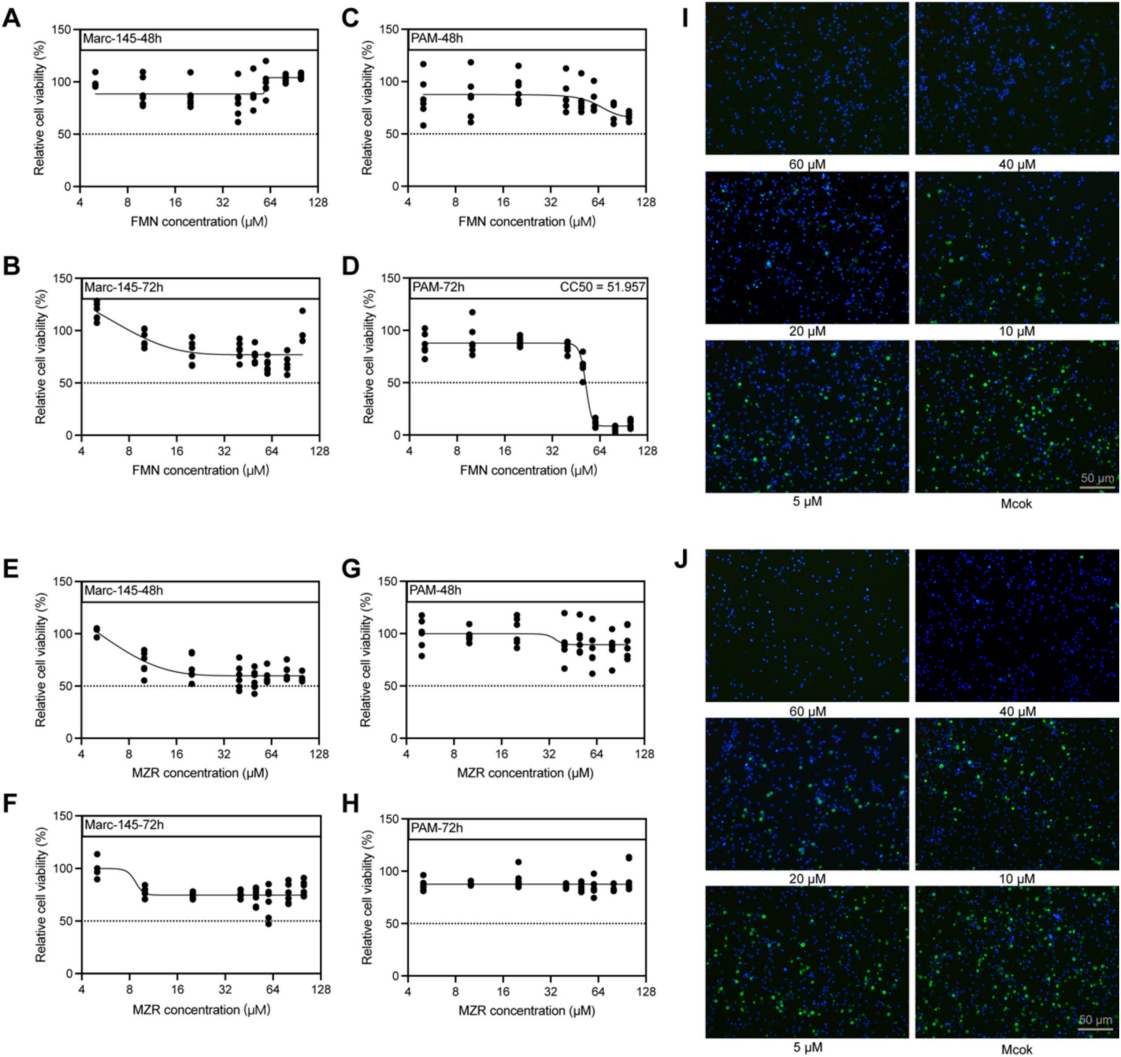
Evaluating the cytotoxicity of FMN and MZR on Marc-145 and PAM Cells. **(A,B)** CC_50_ values for FMN in Marc-145 cells at 48 and 72 h, determined via the CCK-8 assay. **(C,D)** CC_50_ of FMN in PAM cells assessed at 48 and 72 h. **(E,F)** CC_50_ values for MZR in Marc-145 cells at 48 and 72 h. **(G,H)** CC_50_ for MZR in PAM cells, also measured at 48 and 72 h. **(I)** The antiviral activity of FMN against the PRRSV VR-2332 strain, evaluated via IFA. **(J)** Antiviral effectiveness of MZR against the VR-2332 strain, assessed using IFA. Control samples were treated with a matching volume of 0.4% DMSO in DMEM. Black dots within the figure symbolize individual data points from each independent experiment. IFA images are from three experimental replicates.

Our cytotoxicity analysis in Marc-145 cells revealed that administering various concentrations of FMN for both 48 and 72 h resulted in no significant cytotoxic effects (Figures 2A,B). A parallel assessment with MZR under identical conditions similarly indicated minimal cytotoxicity (Figures 2E,F). When examining PAM cells, FMN treatments for 48 h were found to be non-toxic (Figure 2C), while a notable CC_50_ was identified at 51.96 μM after a prolonged exposure of 72 h (Figure 2D). Notably, extended MZR treatment up to 72 h also displayed negligible cytotoxicity in PAM cells (Figures 2G,H). These findings suggest that both FMN and MZR are non-cytotoxic at concentrations below 100 μM and treatment durations shorter than 48 h. Within this identified safety range, we further noted the antiviral efficacy of FMN and MZR against the VR-2332 strain in PAM cells (Figures 2I,J).

### Antiviral activity of FMN and MZR *in vitro*

**Figure 3:**
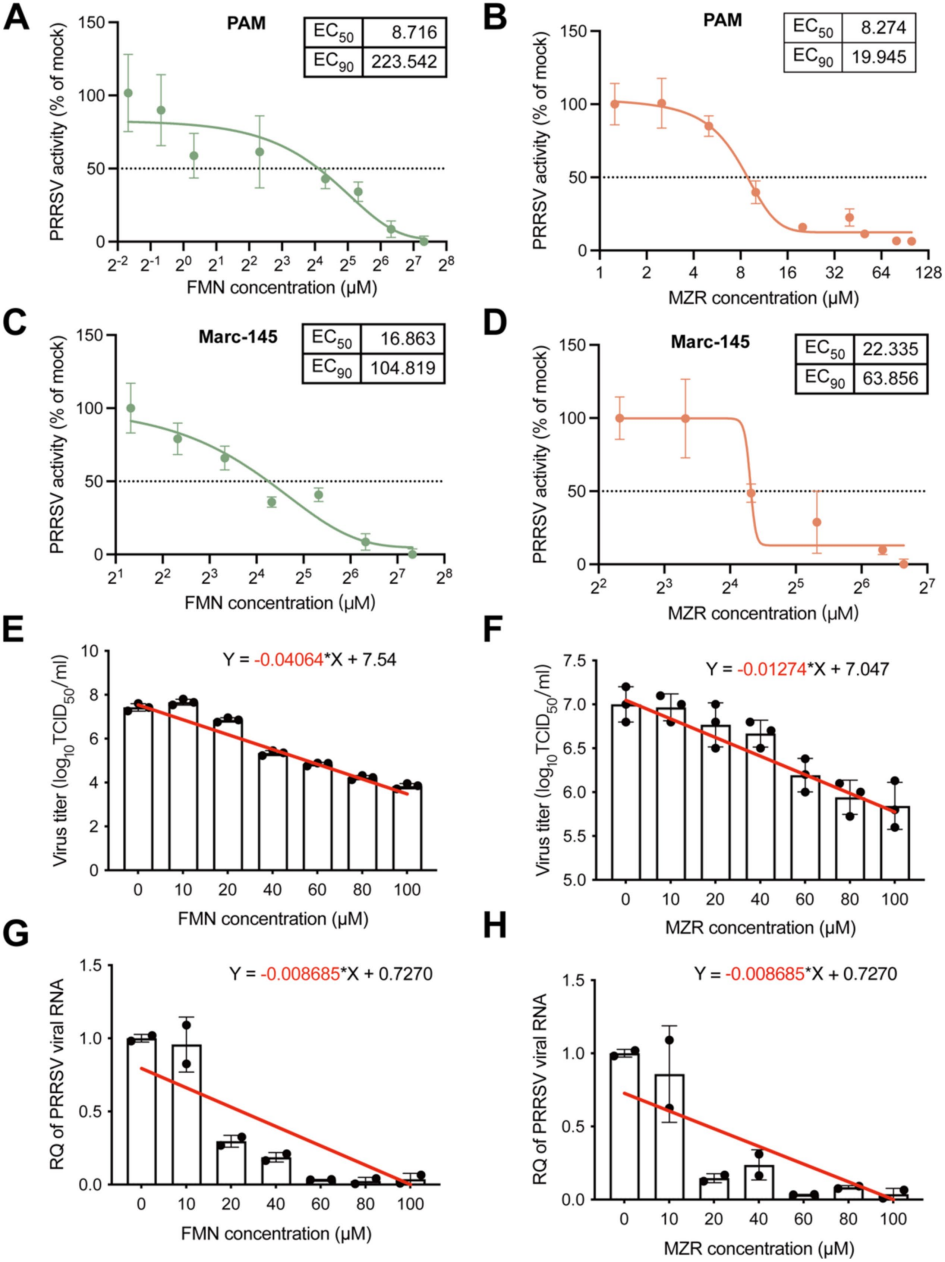
Assessing the antiviral potency of FMN and MZR on PRRSV. This figure delineates the evaluation of FMN and MZR at varying concentrations for their antiviral effects on the VR-2332 strain. **(A,C)** Display the EC_50_ of FMN in PAM and Marc-145 cells, respectively. **(B,D)** Illustrate the EC_50_ of MZR in PAM and Marc-145 cells. **(E,F)** Show alterations in the viral titers of the VR-2332 strain post 36-h incubation with distinct concentrations of FMN or MZR. **(G,H)** Represent the viral genome changes under similar conditions. Each point in the figure corresponds to raw data from individual experiments. Linear fit slopes are marked in red. Error bars indicate the mean ± SD.

This study evaluated the EC_50_ of FMN and MZR against the VR-2332 strain of PRRSV in both Marc-145 and PAM cells. Our findings revealed that FMN’s EC_50_ in PAM cells was approximately 8.72 μM (Figure 3A), and in Marc-145 cells, it was about 16.86 μM (Figure 3C). For MZR, the EC_50_ in PAM cells was roughly 8.27 μM (Figure 3B), while in Marc-145 cells, it approached 22.33 μM (Figure 3D). In the antiviral impact of FMN and MZR on PRRSV, we analyzed viral titers and genomes. The analysis confirmed that the concentrations of FMN and MZR are negatively correlated with the viral titer (slope *m* is negative, Figures 3E,F) and also negatively correlated with the relative expression of the PRRSV genome (slope *m* is negative, Figures 3G,H). This suggests that FMN and MZR inhibit PRRSV replication in a dose-dependent manner, with concentrations above 20 μM effectively suppressing viral replication.

### Effectiveness of FMN and MZR against different PRRSV strains

**Figure 4:**
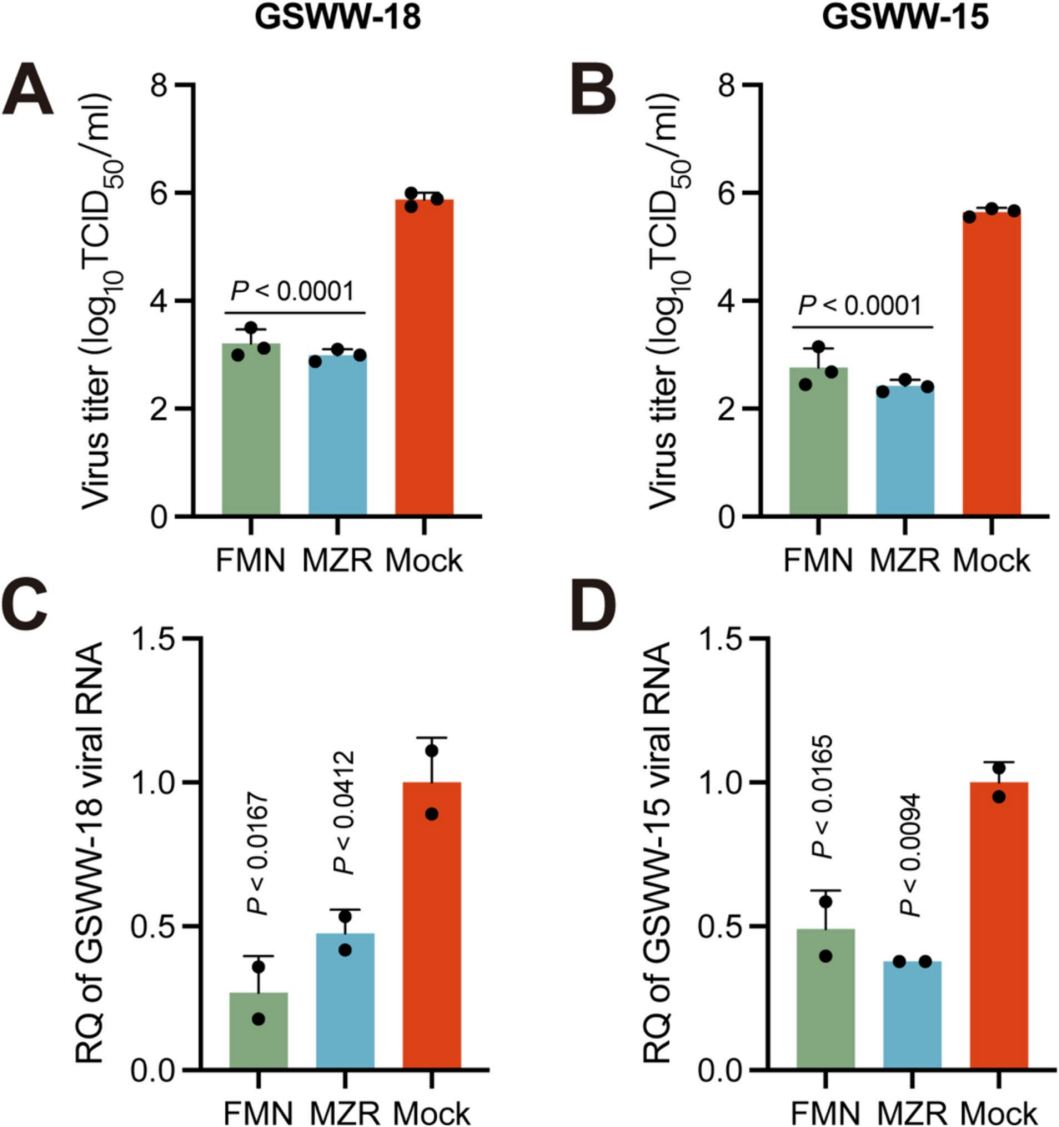
Inhibitory effects of FMN and MZR on GSWW-18 and GSWW-15 Strains. **(A,C)** Changes in PRRSV viral titers in PAM cells incubated with 20 μM of FMN or MZR for 36 h with the GSWW-18 strain. **(B,D)** Changes in PRRSV viral genome in PAM cells after 36 h of incubation with 20 μM of FMN or MZR with the GSWW-15 strain. The black dots in the figure represent the raw data from each independent experiment. *p*-values were calculated using one-way ANOVA with Dunnett’s multiple-comparison test. The *p*-value less than 0.03 was considered statistically significant. Error bars represent the mean ± SD.

In our comprehensive analysis of two additional PRRSV strains maintained in our laboratory, we sought to assess the consistency of antiviral effects of FMN and MZR across diverse strains. In PAM cell assays, both FMN and MZR demonstrated a dose-dependent reduction in the viral titers of the GSWW-18 and GSWW-15 strains (Figures 4A,B). Moreover, we observed a dose-dependent inverse relationship between the concentrations of FMN or MZR and the gene expression levels of these strains (Figures 4C,D). Collectively, these findings corroborate the broad-spectrum antiviral efficacy of FMN and MZR against multiple PRRSV strains.

### FMN and MZR act primarily in the early stages of viral replication

This segment of our study focused on pinpointing the specific phase of the PRRSV lifecycle at which FMN and MZR exert their inhibitory effects. Utilizing a time-of-addition assay, we methodically introduced FMN or MZR to the infected cells at varied time intervals to assess their impact on the virus’s replication cycle (Figure 1A). Notably, introducing FMN or MZR in the initial stages post-infection resulted in pronounced antiviral activity. However, administering lower concentrations of these compounds 8 h post-infection did not significantly impede PRRSV replication (Figure 1B, *P* > 0.1234). Moreover, introducing FMN or MZR 16 h after infection proved entirely ineffective in curtailing viral replication (Figures 1B,C *P* > 0.1234). These observations strongly suggest that FMN and MZR are most effective in inhibiting PRRSV replication during the early stages of the infection process.

### Exploring the synergistic and antagonistic interactions of FMN, MZR, and RBV

**Figure 5:**
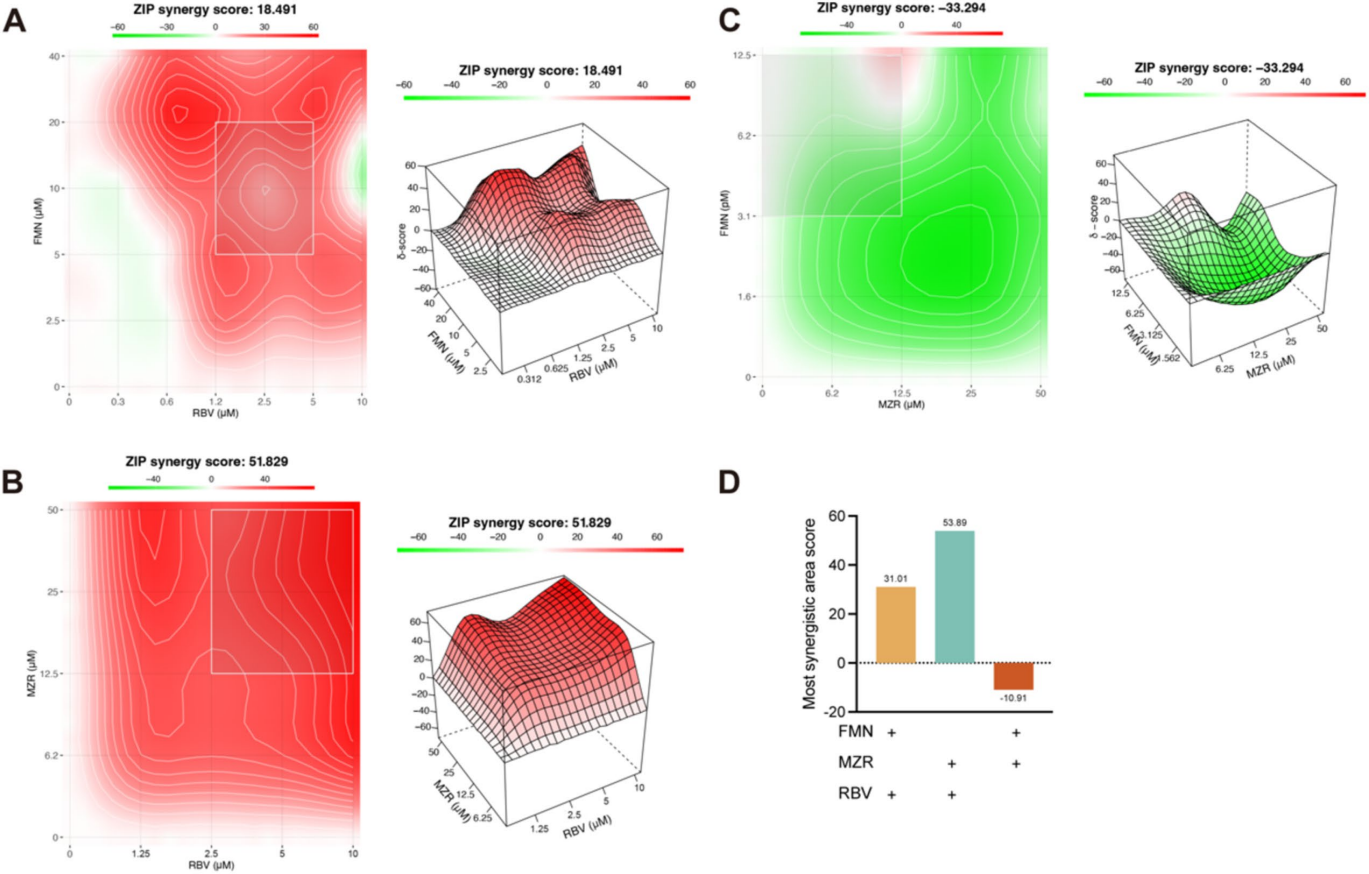
Synergistic interactions of FMN or MZR with Ribavirin. **(A,B)** Analyze the drug combination effects of FMN or MZR with Ribavirin. **(C)** Examines the drug interaction between FMN and MZR. **(D)** Presents a statistical analysis of the most synergistic scores of FMN, MZR, and RBV. The ZIP model in SynergyFinder was employed to analyze and represent the composite effects of these drug interactions. Within this model, the synergy score is the average of all *δ* scores across the dose–response landscape. Synergistic effects are indicated by red areas in the charts, whereas green areas signify antagonistic effects. A ZIP synergy score above 10 is indicative of synergy, while a score below 10 suggests antagonism (24). All experiments were independently conducted three times, and the results of the drug combination analysis were determined using RT-qPCR.

Subsequently, we assessed the impact of FMN, MZR, and RBV through a checkerboard assay. We employed the ZIP model to quantify the interaction scores for various drug concentration combinations. The analysis identified a synergistically optimal region for FMN and RBV (FMN 20 μM–5 μM, and RBV 5 μM–1.25 μM) with a score of 31.01 (Figure 5A), and an average synergy score of 18.49. Similarly, the combination of MZR and RBV demonstrated optimal synergy (MZR 50 μM–12.5 μM, RBV 10 μM–2.5 μM) with a score of 53.89 (Figure 5B) and an average synergy score of 51.83. Contrarily, FMN and MZR together exhibited an antagonistic relationship, reflected by a score of −33.29 (Figure 5C). These results suggest that while FMN or MZR synergize effectively with RBV, their combination displays antagonism (Figure 5D).

## Discussion

In the early twentieth century, review articles concerning antiviral chemotherapy in veterinary medicine highlighted several disadvantages associated with the use of antiviral drugs in this field (26). These issues encompassed limited applicability to specific viruses and animal species, difficulty in achieving high activity with low cytotoxicity, elevated costs in developing new compounds, and a deficiency in rapid diagnostic techniques permitting the quick use of specific antiviral drugs during acute disease courses (26). Most antiviral drugs utilized in veterinary medicine were originally developed for human viral infections, and their clinical application in veterinary medicine remains limited (27).

Our current focus is to identify strategies to control the PRRSV pandemic beyond vaccine immunization. However, the critical and irreplaceable role of vaccine immunization in controlling pandemics cannot be denied (28). Therefore, our approach involves employing antiviral drugs in regions with high prevalence to control the pandemic, aiming to aid farms in rapidly transitioning to a positive or stable negative status, thus acting as a supplementary measure to vaccine immunization. FMN, which is extracted from the inflorescences, flower-bearing stems, and leaves of the leguminous plant red clover, exhibits a wide range of biological functions (29). Natural products offer advantages such as multiple targets, multiple pathways, and fewer adverse reactions (30). MZR, an imidazole antibiotic derived from the culture of soil fungus (*Eupenicillium brefeldianum*), falls within the class of imidazole nucleosides (31). MZR, functioning as an immunosuppressant, is widely used in treating conditions such as kidney transplant rejection, lupus nephritis, rheumatoid arthritis, and nephrotic syndrome, as well as other autoimmune diseases (32).

In our previous studies, FMN and MZR were identified as effective inhibitors of FCV proliferation *in vitro* (17, 18). This research extends those findings to evaluate their antiviral effects against PRRSV. Our results confirm the efficacy of both FMN and MZR in inhibiting PRRSV proliferation. Notably, Marc-145 cells treated with FMN or MZR for 72 h showed reduced CC_50_ values, while treatments under 48 h exhibited minimal cytotoxicity. Significantly, this study first reports the inhibitory effects of FMN and MZR on PRRSV in PAM cells. We demonstrated that these compounds effectively inhibit PRRSV strains VR-2332, GSWW-15, and GSWW-18, with notably low EC_50_ values in PAM cells, suggesting their potential clinical utility. Previous research has highlighted FMN’s robust antiviral activity against viruses like EV71 and SARS-CoV-2 (33, 34). As a FGFR2 inhibitor, the relationship between FMN’s antiviral properties and this inhibition warrants further exploration (35). Interestingly, while FMN and MZR exhibit antagonistic effects when combined, both show synergistic effects with RBV. This suggests that FMN and MZR may share antiviral targets, albeit with mechanisms distinct from RBV. MZR, as an IMPDH inhibitor akin to Ribavirin, displays antiviral activity against a range of viruses, including CpHV-1, FMDV, SARS-Cov, and BVDV (36–39). Our observations indicate that FMN and MZR predominantly act during the early stages of PRRSV infection, potentially due to MZR’s inhibition of *de novo* purine synthesis, which impedes RNA replication in early PRRSV replication. Additionally, FMN has been shown to inhibit EV-A71-induced phosphorylation of ERK, p38, and JNK, thereby suppressing viral replication by targeting cellular inflammatory pathways (40). Further research is needed to elucidate FMN’s specific antiviral targets. Given PRRSV’s reliance on cellular inflammatory pathways for replication, it is plausible that FMN could also hinder its proliferation by mitigating PRRSV-induced cellular inflammation. The antiviral mechanisms of FMN and MZR are compelling, and their further exploration could lay the groundwork for novel antiviral drug development.

In pig farming, Porcine Reproductive and Respiratory Syndrome (PRRS) ranks as a prevalent viral disease (4). Currently, the efficacy of veterinary drugs and vaccines in clinical use falls short of expectations, making the effective control of PRRSV appear elusive (41, 42). Our study suggests that FMN and MZR hold promise as inhibitors of PRRSV. We have ascertained that higher doses of FMN or MZR correlate with significant antiviral activity against PRRSV. Notably, the primary antiviral action of these compounds manifests in the early stages of viral infection. In future studies, the efficacy of FMN or MZR in pigs and their pharmacokinetic parameters should be confirmed to provide a basis for clinical applications. Given these findings, it becomes imperative to conduct thorough research on the potential resistance to FMN and MZR. Conclusively, with their demonstrated *in vitro* antiviral effectiveness, FMN and MZR could emerge as pivotal elements in the strategic management of PRRSV, potentially altering the landscape of future pandemic responses.

## Data Availability

The raw data supporting the conclusions of this article will be made available by the authors, without undue reservation.
